# The Presence of an In Situ Component on Pre-Treatment Biopsy Is Not Associated with Response to Neoadjuvant Chemotherapy for Breast Cancer

**DOI:** 10.3390/cancers13020235

**Published:** 2021-01-10

**Authors:** Julie Labrosse, Charlotte Morel, Thanh Lam, Enora Laas, Jean-Guillaume Feron, Florence Coussy, Marick Lae, Fabien Reyal, Anne-Sophie Hamy

**Affiliations:** 1 Department of Surgery, Institut Curie, 75005 Paris, France; julie.labrosse@aphp.fr (J.L.); charlotte.morel@aphp.fr (C.M.); enora.laas@curie.fr (E.L.); jean-guillaume.feron@curie.fr (J.-G.F.); 2 Department of Gynecology and Obstretrics, Hôpitaux Universitaires de Genève, 1205 Geneva, Switzerland; Giang.T.Lam@hcuge.ch; 3 Medical Oncology Department, Centre René Hughenin, 92210 Saint Cloud, France; florence.coussy@curie.fr (F.C.); anne-sophie.hamy-petit@curie.fr (A.-S.H.); 4 Department of Tumor Biology, Institut Curie, 75005 Paris, France; marick.lae@curie.fr; 5 Residual Tumor & Response to Treatment Laboratory, RT2Lab, Translational Research Department, INSERM, U932 Immunity and Cancer, 75248 Paris, France

**Keywords:** breast cancer, neoadjuvant chemotherapy (NAC), ductal carcinoma in situ (DCIS), pathological complete response (pCR)

## Abstract

**Simple Summary:**

Identifying markers predictive of response and resistance to neoadjuvant chemotherapy (NAC) has become a major research objective. Ductal carcinoma in situ (DCIS) is associated with invasive disease in more than half of invasive breast cancer cases. It is generally assumed that DCIS does not respond to NAC, but the effect of chemotherapy on in situ components has been little studied. We assessed the predictive value of the presence of an in situ component on pre-NAC biopsy on pathological complete response (pCR) in a real-life cohort of patients treated by NAC. We included 1148 patients; 44% of tumors were luminal (*n* = 508), 31% triple negative breast cancer (TNBC) (*n* = 359) and 24% *HER2*-positive (*n* = 281). DCIS was found in 225 samples (19.6%) before NAC. The presence of a DCIS component on pre-NAC biopsy was not associated with pCR and did not seem to be a critical factor for the prediction of response to NAC.

**Abstract:**

A ductal in situ (DCIS) component is often associated with invasive breast carcinoma (BC), and its effect on response to treatment is unknown. We assessed the predictive value of the DCIS component for pathologic complete response (pCR) after neoadjuvant chemotherapy (NAC). We analyzed a cohort of 1148 T1–3NxM0 breast cancer (BC) patients treated by NAC at Institut Curie between 2002 and 2012. The presence of a DCIS component was retrospectively recorded from both the pre-NAC biopsy pathological report and surgical specimens. We included 1148 BC patients treated by NAC for whom pre- and post-NAC data concerning the in situ component were available. DCIS was present before NAC in 19.6% of the population. Overall, 283 patients (19.4%) achieved pCR after NAC. There was no significant association between the presence of DCIS on pre-NAC biopsy and pCR. In a multivariate analysis including subtype, tumor size, grade, mitotic index, and Ki67 index, only BC subtype (luminal/TNBC/*HER2*-positive) and Ki67 were significantly associated with pCR. The presence of a DCIS component on pre-NAC biopsy is not associated with pCR and does not seem to be a critical factor for predicting response to NAC.

## 1. Introduction

Neoadjuvant or pre-operative chemotherapy (NAC) is administered to patients with inflammatory or locally advanced breast cancer (BC). It is widely used, even for early-stage BC. Its ability to reduce the size of some tumors, rendering them resectable [1,2], increases the rate of breast-conserving surgery rates. It also makes it possible to analyze the effect of chemotherapy on the tumor itself, through evaluations of residual tumor burden on surgical specimens. Furthermore, patients achieving a pathological complete response (pCR) after neoadjuvant systemic treatment have been shown to have better long-term outcomes [3,4]. The identification of markers predictive of response and resistance to NAC has become an important research objective [5]. The major clinical and biological factors shown to date to be predictive of the chances of achieving pCR are age [6], body mass index (BMI) [7], levels of the proliferation biomarker Ki67 [8], estrogen receptor status [5], and, more recently, levels of tumo-infiltrating lymphocytes (TILs) [9].

Ductal carcinoma in situ (DCIS) is associated with invasive disease in more than half of invasive BC cases [10]. It is defined as a neoplasic proliferation of epithelial cells confined to the ductal-lobular system that can evolve to invasive BC, having biological specificities [11,12,13]. Indeed, invasive breast carcinoma associated to a DCIS component has been reported have biological specificities, with a proper immune environment, including TILs and immune cells [11]. Furthermore, invasive BC with a DCIS component have also been associated to lower proliferation rates and lower metastatic potential compared to pure invasive carcinoma, notably when the proportion of DCIS to invasive carcinoma is high [10]. While some studies show that DCIS does not respond to NAC [14], others suggest that DCIS components might completely be eradicated after NAC, notably in *HER2*-positive tumors [15,16], and that there exists a strong correlation between invasive and non-invasive components in terms of pCR [17]. Altogether, despite its clinical relevance, whether the presence of a DCIS component in invasive breast cancer could modify response to NAC has scarcely been studied.

We assessed the predictive value of the presence of an in situ component on pre-NAC biopsy for pCR in a real-life cohort of patients treated by NAC.

## 2. Results

### 2.1. Patients and Tumor Characteristics

In total, 1148 patients were included in the cohort. Most were premenopausal (63%, *n* = 713), and 13% (*n* = 146) were obese (BMI > 30). Clinically, most patients had stage T2 tumors (67%, *n* = 764) and node-positive BC (56%, *n* = 644); 44% of tumors were luminal (*n* = 508), 31% were TNBC (*n* = 359) and 24% were *HER2*-positive BC (*n* = 281), including 134 *HER2*+/ER− and 147 *HER2+*/ER+. Our cohort comprised 281 *HER2*-positive tumors, of which 43 (15.3%) were diagnosed before 2005 and 238 (84.7%) after 2005; 198 (70.5%) received neoadjuvant trastuzumab. Most tumors were grade 3 (59.1%, *n* = 659). A DCIS component was present before NAC in 19.6% of samples (*n* = 225) (Table 1).

### 2.2. Pre-NAC DCIS

**Figure 1:**
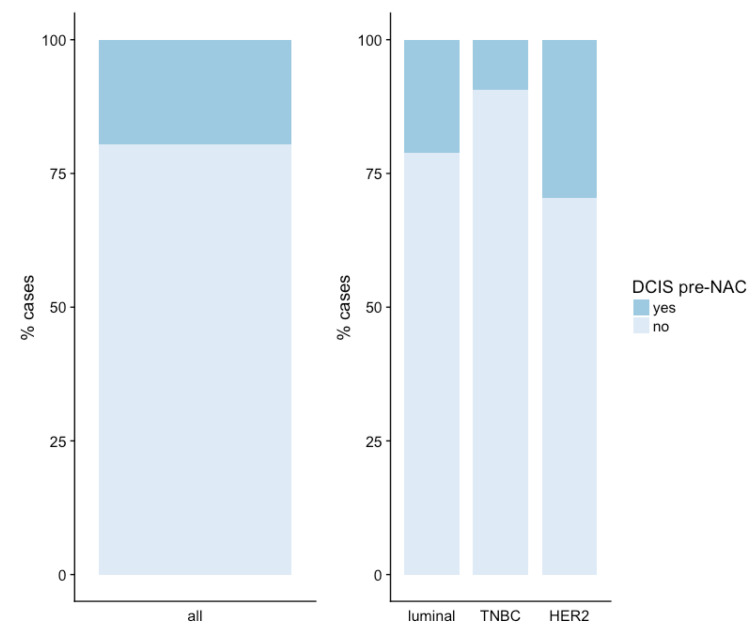
Presence of a ductal carcinoma in situ (DCIS) component on breast cancer biopsy before neoadjuvant chemotherapy (NAC). Abbreviations: ductal carcinoma in situ (DCIS), neoadjuvant chemotherapy (NAC).

DCIS was found in 225 samples obtained before NAC (19.6%). The presence of a DCIS component was associated with BC subtype (*p* < 0.001). The percentage of pre-NAC samples with a DCIS component was higher for *HER2*-positive BC (29.5%) than for luminal BC (21.3%) or TNBC (9.5%) (Figure 1).

The presence of a DCIS component on pre-NAC biopsy was associated with menopausal status, BMI, mitotic index and grade (Table 2). Most of the pre-NAC samples with a DCIS component came from patients who were premenopausal (73.2%), with a BMI ≤ 30 (92.9%); most of the tumors were grade I–II (51.4%), with a lower mitotic index (75.1%).

### 2.3. Post-NAC DCIS

**Figure 2:**
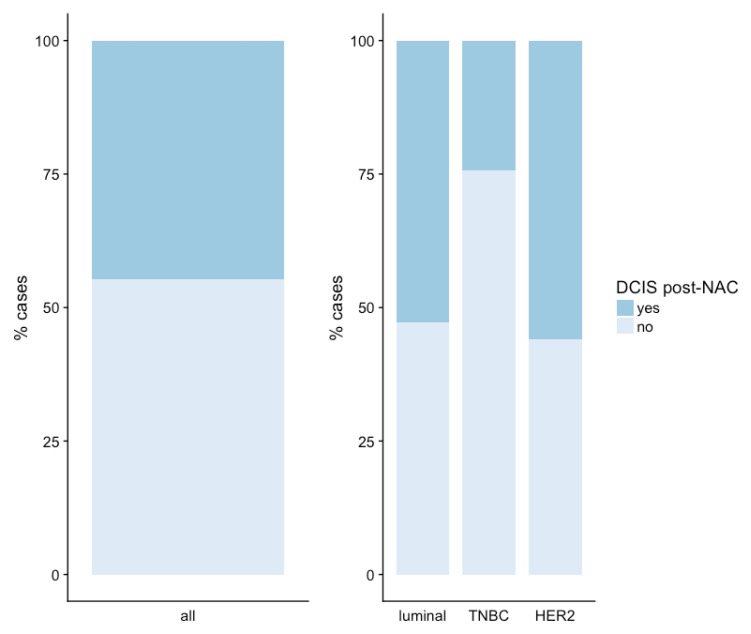
Presence of a DCIS component in post-NAC breast cancer surgical specimens.

The proportion of post-NAC surgical specimens with a DCIS component depended on BC subtype: 54.4% for *HER2*-positive BC, 53.3% for luminal BC, 24% for TNBC, *p* < 0.001 (Figure 2).

### 2.4. Change in the DCIS Component between the Pre- and Post-NAC Biopsies

Paired pre- and post-NAC data concerning the presence of DCIS were available for 1148 patients (508 luminal, 359 TNBC and 281 *HER2*-positive BC).

**Figure 3:**
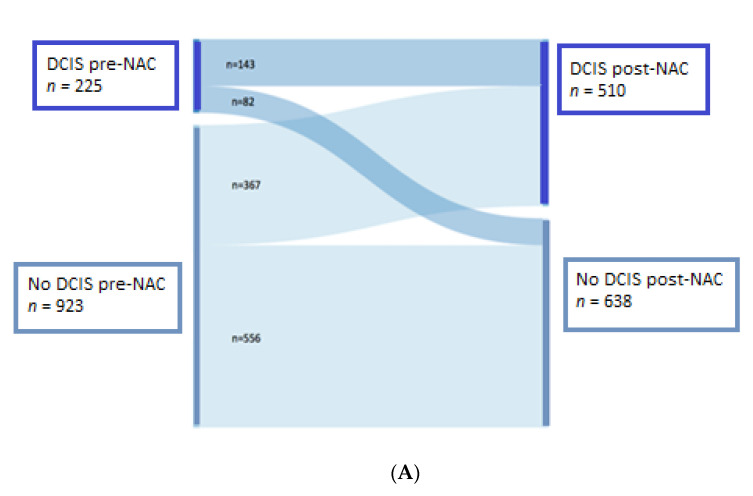

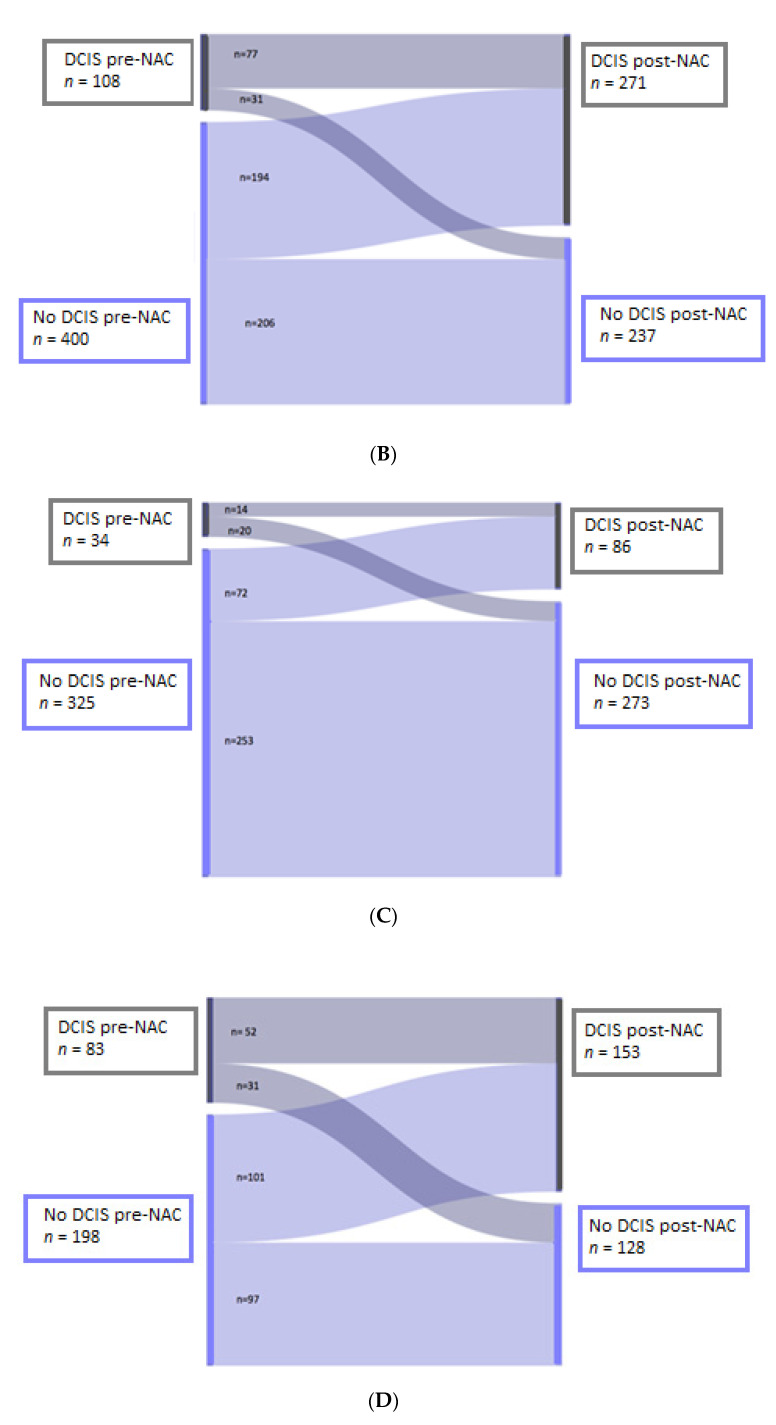
Presence of ductal carcinoma in situ (DCIS) component before and after neaoadjuvant chemotherapy (NAC) (**A**) Presence of DCIS before and after NAC, for the total population (1148 patients). (**B**) Presence of DCIS before and after NAC for luminal BC (508 patients). (**C**) Presence of DCIS before and after NAC for TNBC (359 patients). (**D**) Presence of DCIS before and after NAC for *HER2*-positive BC (281 patients). Abbreviations: ductal carcinoma in situ (DCIS), neoadjuvant chemotherapy (NAC).

DCIS was present in both the microbiopsy and surgical specimens for 143 patients. In 556 cases, no DCIS was detected either pre- or post-NAC (Figure 3A–D). In 367 cases (32%), no DCIS was detected in the pre-NAC specimen, but a DCIS component was present in the surgical specimen after NAC. For 82 patients, DCIS was present in the pre-NAC specimen but not in the surgical specimen.

In the total population, the proportion of samples displaying a DCIS component was significantly higher after than before NAC (44.4% versus 19.6%). Similar results were obtained for the various BC subtypes (*HER2*-positive BC: 54.4% versus 29.5% (*p =* 0.002), luminal: 53.3% versus 21.3% (*p* < 0.0001), TNBC: 24% versus 9.5% (*p <* 0.0001)), (Table 3).

In the total population, the percentage of samples with a DCIS component was 19.6% before NAC and 44.4% after NAC (*p* < 0.0001). For luminal tumors, the percentage of samples with a DCIS component was 21.3% before NAC and 53.3% after NAC (*p* < 0.0001). The corresponding proportions were 9% before NAC and 24% after NAC for TNBC (*p* < 0.0001) and 29.5% before NAC and 54.4% after NAC for *HER2* tumors (*p* = 0.002).

DCIS was detected on pre-NAC biopsy in 46 of the patients who achieved (16.3%), distributed as follows: 7 luminal BCs (21.9%), 10 TNBC s (7.1%) and 29 *HER2*-positive BCs (26.4%). We found that 179 (20.7%) of the patients who did not achieve pCR had a DCIS component in the pre-NAC specimen: 101 luminal (21.2%), 24 TNBC (11%) and 54 *HER2*-positive BCs (31.6%).

### 2.5. Baseline Clinical and Pathological Parameters Associated with pCR

Overall, 283 patients achieved pCR (24.7%) after NAC. In univariate analysis, the baseline clinical and pathological factors significantly associated with higher pCR rates were (Table 4): TNBC or *HER2*-positive BC subtype, high grade, high mitotic index and Ki67 ≥ 20. Tumor size ≥T2 was associated with lower rates of pCR.

In a multivariable logistic regression analysis including subtype, tumor size, grade, mitotic index and Ki67 index, only subtype and Ki67 were significantly associated with pCR.

The presence or absence of DCIS on biopsy was not significantly associated with response to NAC. Indeed, the pCR rate was 20.4% in cases of DCIS on the pre-NAC biopsy vs. 25.7% for cases with no DCIS on the pre-NAC biopsy (OR = 0.74 (95% CI: 0.52–1.06), *p =* 0.1).

## 3. Discussion

In this study, no significant association was found between the presence of DCIS on pre-NAC biopsy and histological response to NAC.

The proportion of samples with an in situ component associated with an invasive component (19.6%) was lower than reported in other cohorts. Depending on the study considered, the proportion of tumors with an in situ component adjacent to invasive BC ranges from 33% [15] to 53% [10]. This may reflect lower rates of in situ disease in more advanced BC treated by NAC. In addition, the real rate of pre-NAC DCIS may have been underestimated due to the small amount of tissue collected in pre-treatment biopsies.

The percentage of pre-NAC samples displaying in situ disease was higher for *HER2*-positive BC (29.5%) than for luminal BC (21.3%) or TNBC (9.5%), (*p* < 0.001). Consistently, Doebar et al. [18] found that DCIS was significantly more frequent in *HER2*-positive tumors compared to luminal or TNBC. However, to our knowledge, no other study has ever assessed the presence of DCIS, as a function of BC subtype, on biopsy specimens before treatment.

On post-NAC surgical specimens, the proportion of samples with a DCIS component was higher for *HER2*-positive BC (54.4%) than for luminal BC (53.3%) or TNBC (24%). Wong et al. reported similar postoperative results in a study on 1159 patients with BC of no specific type (NST) treated by upfront surgery [19]. They found that DCIS was associated with NST carcinoma in 63.2% of *HER2*-positive BCs, versus 53.3% of luminal BCs and 33.3% of TNBCs.

In our study, DCIS was present in the pre-NAC specimen but not in the surgical specimen for 82 patients. This finding may be accounted by the in situ component being present, in its entirety, in the biopsy specimen, or by chemotherapy having an effect on DCIS. Given the probable underestimation of the real rate of adjacent DCIS on pre-NAC biopsy specimens, it seems likely that the frequency of complete DCIS eradication was also underestimated. The response of adjacent DCIS to NAC has been described in a few studies. Goldberg et al. [15] investigated the impact of neoadjuvant chemotherapy on DCIS in a cohort of 92 patients with locally advanced BC and found that NAC +/− trastuzumab was able to eradicate the in situ component completely. Indeed, both the invasive and non-invasive components disappeared in 33% of the patients in the trial concerned. Matsuo et al. [17] reported pathological response to be strongly correlated between invasive and non-invasive components in a series of 100 primary BCs treated by NAC. In Von Minckwitz et al.’s analysis of 158 *HER2*-positive breast cancer patients treated by NAC combined with trastuzumab [16], 50.8% of the samples in which a DCIS component was associated with invasive carcinoma showed a complete eradication of DCIS after NAC.

For 367 paired samples (32% of the pairs), there was no DCIS pre-NAC, but an in situ component was detected in the surgical specimen after NAC. These paired samples should probably be considered “false negatives” due to a lack of representativeness of the pre-NAC biopsy specimen.

pCR was achieved for 283 patients (24.7%). No significant association between the presence or absence of a pre-NAC DCIS component and pCR was observed. Consistently, Van Bockstal et al. [20] found no association between a DCIS component and pCR in a cohort of TNBC patients. By contrast, Von Minckwitz et al. [16] found that the presence of DCIS associated with *HER2*-positive breast cancer was an independent negative predictor of pCR after NAC (OR = 0.42 (95% CI 0.2–0.9), *p =* 0.0027). However, whereas about two thirds of *HER2*-positive tumors in our cohort had received neoadjuvant trastuzumab, all patients had been treated by an anthracycline-taxane-trastuzumab NAC regimen. Ki67 has also been investigated as a predictive marker of pCR after NAC, and 20% ki67 has been suggested as the most relevant cut-off value to distinguish pCR from absence of response [21].

## 4. Materials and Methods

### 4.1. Patients

We analyzed a cohort of 1148 T1-3NxM0 patients with invasive breast carcinoma (NEOREP Cohort, CNIL declaration number 1547270) treated at Institut Curie, Paris, between 2002 and 2012. We included only patients with unilateral, non-recurrent, non-inflammatory, non-metastatic tumors with an indication of NAC, for whom pre- and post-NAC data were available for the DCIS component. All patients received NAC, followed by surgery and radiotherapy when indicated. The study was approved by the Breast Cancer Study Group of Institut Curie, and the CNIL gave its authorization for data analysis for research purposes. This study was conducted according to institutional and ethics committee rules regarding research on tissue specimens and patients.

### 4.2. Treatments

Patients were treated according to national guidelines. NAC regimens changed over time (anthracycline-based regimen or sequential anthracycline-taxane regimen), with trastuzumab used in an adjuvant and/or neoadjuvant setting since 2005 for *HER2*-positive breast cancer. Surgery was performed four to six weeks after the end of chemotherapy. Most patients (98.2%, *n* = 1127) received adjuvant radiotherapy. Endocrine therapy (tamoxifen, aromatase inhibitor, and/or GnRH agonists) was prescribed when indicated.

### 4.3. Tumor Samples

Tumor samples were collected in routine care for the management of breast cancer at Institut Curie. ER and PR statuses were determined as follows. Tissue sections were rehydrated and antigen retrieval was performed in citrate buffer (10 mM, pH 6.1). The sections were then incubated with antibodies against ER (clone 6F11, Novocastra, Leica Biosystems, Newcastle, UK; 1/200) and PR (clone 1A6, Novocastra, 1/200). Antibody binding was detected with the Vectastain Elite ABC peroxidase-conjugated mouse IgG kit (Vector, Burlingame, CA, USA), with diaminobenzidine (Dako A/S, Glostrup, Denmark) as the chromogen. Positive and negative controls were included in each run. In accordance with French recommendations, cases were considered positive for ER or PR if at least 10% of the tumor nuclei were stained [22]. Tumors were considered hormone receptor (HR)-positive if they were positive for either ER or PR, and HR-negative when negative for both ER and PR.

Mitotic index was assessed using a microscope with an objective of field diameter = 0.62 mm (×40 objective). Mitotic cells were counted on 10 high-power fields (HPF) (1 HPF = 0.301 mm^2^). Cutoffs of <11, 12–22 and >22 mitoses were used to define low, intermediate and high mitotic indices, respectively, according to the international recommendations [23].

For Ki-67 assessment, tissue sections were incubated for one hour with an anti-Ki67 monoclonal antibody (Clone MIB1, Dako A/S, Glostrup, Denmark) at a dilution of 1/100. Staining was detected with the Vectastain Elite ABC peroxidase mouse IgG kit (Vector Burlingame, CA, USA), with diaminobenzidine (Dako A/S) as the chromogen. The semiquantitative assessment was performed by estimating, at ×200 magnification, the percentage of positive neoplastic nuclei within the area of highest positivity chosen on scanning of the entire tumor area at low power (×10 objective) [24]. All nuclei with homogeneous staining, even if only light or exclusively nucleoli, were considered to be positive.

*HER2* expression was determined by immunohistochemistry with a monoclonal anti-*HER2* antibody (CB11, Novocastra, New-Castle, UK; 1/800). Scoring was performed according to American Society of Clinical Oncology (ASCO)/College of American Pathologists (CAP) guidelines [25]. Scores of 3+ were considered positive, and scores of 1+/0 were considered negative. Tumors with scores of 2+ were subjected to FISH with a *HER2* gene-specific probe and a centromeric probe for chromosome 17 (PathVysion *HER-2* DNA Probe kit, Vysis-Abbott, Abbott Park, IL, USA), in accordance with the kit manufacturers’ instructions. *HER2* gene amplification was defined according to ASCO/CAP guidelines [13]. We evaluated a mean of 40 tumor cells per sample and the mean *HER2* signal per nucleus was calculated. A *HER2*/CEN17 ratio ≥ 2 was considered positive, and a ratio < 2 was considered negative [13].

The presence or absence of a DCIS component was retrospectively extracted from the pathology reports for pretreatment core needle biopsy and surgical specimens. The presence of a DCIS component was considered as a binary variable (yes/no), and all tumor samples containing a DCIS component were considered, regardless of subtype.

### 4.4. Study Endpoints

The ypTN stage of the tumors was determined according to the American Joint Committee on Cancer/Union for International Cancer Control staging. A pathological complete response (pCR) was defined as the absence of invasive residual tumor in the breast and axillary nodes (ypT0/is+ ypN0).

### 4.5. Statistical Analysis

The study population was described in terms of frequencies for qualitative variables, or medians, means and associated ranges for quantitative variables. Comparisons of the proportion of samples with a DCIS component before and after NAC were performed with McNemar tests.

Factors predictive of pCR were introduced into a univariate logistic regression model. A multivariate logistic model was then implemented. The covariates selected for multivariate analysis were those with a *p*-value for the likelihood ratio test below 0.05 in univariate analysis.

A significance threshold of 5% was used. Analyses were performed with R software, version 3.1.2 (RStudio Team (2018). Rstudio: Integrated Development for R. RStudio, Inc., Boston, MA, USA, URL http://www.rstudio.com).

## 5. Conclusions

Overall, the presence of a DCIS component on pre-NAC biopsy was not associated with pCR and did not seem to be crucial for the prediction of response to NAC. Further studies are awaited, to validate pre-NAC biomarkers that could potentially improve the prediction of response to neoadjuvant treatment.

## Figures and Tables

**Table 1:** Patients and tumor characteristics before neoadjuvant chemotherapy (NAC).

Variables	Characteristics	*n*	%
Menopausal status	Postmenopausal	426	(37.4%)
	Premenopausal	713	(62.6%)
BMI	BMI < 19	68	(6%)
	BMI: 19 to 25	644	(56.4%)
	BMI: 25 to 30	284	(24.9%)
	BMI > 30	146	(12.8%)
Tumor size	T1	65	(5.7%)
	T2	764	(66.6%)
	T3	318	(27.7%)
Nodal status	N0	503	(43.9%)
	N1–N2–N3	644	(56.1%)
Mitotic index	≤22	684	(64.7%)
	>22	374	(35.3%)
Histology	NST	1022	(89.8%)
	Other	116	(10.2%)
Subtype	Luminal	508	(44.2%)
	TNBC	359	(31.3%)
	*HER2*	281	(24.5%)
Grade	Grade I	46	(4.1%)
	Grade II	411	(36.8%)
	Grade III	659	(59.1%)
KI67	<20	168	(30.3%)
	≥20	387	(69.7%)
DCIS component	No	923	(80.4%)
	Yes	225	(19.6%)

Abbreviations: body mass index (BMI), ductal carcinoma in situ (DCIS), no specific type (NST), Human Epidermal Growth Factor Receptor-2 (*HER2)*. Missing data: menopausal status: 9, BMI: 6, tumor size: 1, nodal status: 1, mitotic index: 90, histology: 10, subtype: 0, grade: 32, ki67: 593, DCIS: 0.

**Table 2:** Association between the characteristics of patients and tumors and the presence of pre-NAC DCIS.

Variables	Characteristics	No DCIS Pre-NAC	DCIS Pre-NAC	*p*
Menopausal status	Premenopausal	549 (60%)	164 (73.2%)	<0.001
	Postmenopausal	366 (40%)	60 (26.8%)	
BMI	BMI < 19	502 (54.6%)	142 (63.4%)	0.007
	BMI: 19 to 25	52 (5.7%)	16 (7.1%)	
	BMI: 25 to 30	234 (25.5%)	50 (22.3%)	
	BMI > 30	130 (14.2%)	16 (7.1%)	
Tumor size	T1	52 (5.6%)	13 (5.8%)	0.79
	T2	619 (67.1%)	145 (64.7%)	
	T3	252 (27.3%)	66 (29.5%)	
Nodal status	N0	407 (44.1%)	96 (42.7%)	0.75
	N+	515 (55.9%)	129 (57.3%)	
Mitotic Index	≤22	530 (62.1%)	154 (75.1%)	0.001
	>22	323 (37.9%)	51 (24.9%)	
Subtype	Luminal	400 (43.3%)	108 (48%)	<0.001
	TNBC	325 (35.2%)	34 (15.1%)	
	*HER2*	198 (21.5%)	83 (36.9%)	
Grade	Grade I–II	346 (38.4%)	111 (51.4%)	0.001
	Grade III	554 (61.6%)	105 (48.6%)	
Ki67	ki67 < 20	120 (28.6%)	48 (35.3%)	0.17
	ki67 ≥ 20	299 (71.4%)	88 (64.7%)	

Abbreviations: body mass index (BMI), ductal carcinoma in situ (DCIS). Missing data: menopausal status: 9, BMI: 6, tumor size: 1, nodal status: 1, mitotic index: 90, subtype: 0, grade: 32, ki67: 593.

**Table 3:** Change in the proportion of samples presenting a DCIS component between the pre- and post-NAC specimens.

Presence or Absence of DCIS		Pre-NAC	Post-NAC
Total population	No DCIS	923 (80.4%)	638 (55.6%)
	DCIS	225 (19.6%)	510 (44.4%)
Luminal	No DCIS	400 (78.7%)	237 (46.7%)
	DCIS	108 (21.3%)	271 (53.3%)
TNBC	No DCIS	325 (90.5%)	273 (76%)
	DCIS	34 (9.5%)	86 (24%)
*HER2*	No DCIS	198 (70.5%)	128 (45.6%)
	DCIS	83 (29.5%)	153 (54.4%)

**Table 4:** Association of baseline clinical and pathological factors with pCR.

Variable		*n*	pCR	%	Univariate Analysis			Multivariate Analysis		
					OR	95% CI (OR)	*p*	OR	95% CI (OR)	*p*
Menopausal	Post	426	113	26.5	1					
status	Pre	713	167	23.4	0.85	0.64–1.12	0.2		–	–
BMI	19–25	644	164	25.5	1					
	<19	68	13	19.1	0.69	0.35–1.26	0.25		–	–
	>25	430	103	24	0.92	0.69–1.22	0.57			
Tumor size	T1	65	32	49.2	1					
	T2	764	186	24.3	0.33	0.20–0.56	<0.0001		–	–
	T3	318	65	20.4	0.26	0.15–0.46	<0.0001		–	–
Nodal status	N0	503	120	23.9	1					
	N1–N2–N3	644	163	25.3	1.08	0.82–1.42	0.57		–	–
Mitotic index	≤22	684	127	18.6	1					
	>22	374	133	35.6	2.42	1.82–3.22	<0.0001		–	–
Histology	Non specific type (NST)	1022	265	25.9	2.04	1.2–3.47	0.01		–	–
	Other	116	17	14.7	1					
Grade	I–II	457	55	12	1					
	III	659	221	33.5	3.69	2.66–5.1	<0.0001		–	–
Ki67	<20	168	11	6.5	1			1		
	≥20	387	105	27.1	5.3	2.77–10.2	<0.0001	3	1.31–7.75	0.01
DCIS	No	923	237	25.7	1					
	Yes	225	46	20.4	0.74	0.52–1.06	0.1			
Subtype	Luminal	508	32	6.3	1			1		
	TNBC	359	141	39.3	9.62	6.43–14.8	<0.0001	5.4	2.68–11.3	<0.0001
	*HER2*	281	110	39.1	9.57	6.29–14.9	<0.0001	8.7	4.39–18.3	<0.0001

Abbreviations: neoadjuvant chemotherapy (NAC), body mass index (BMI), ductal carcinoma in situ (DCIS), pathological complete response (pCR); no specific type (NST).

## Data Availability

The data presented in this study are available on request from the corresponding author.
